# Physiological and transcriptomic analyses to reveal underlying phenolic acid action in consecutive monoculture problem of *Polygonatum odoratum*

**DOI:** 10.1186/s12870-021-03135-x

**Published:** 2021-08-07

**Authors:** Xianzhi Ni, Chenzhong Jin, Aiyu Liu, Yong Chen, Yihong Hu

**Affiliations:** 1 Collaborative Innovation Center for Field Weeds Control of Hunan Province, Hunan University of Humanities, Science and Technology, Loudi, 417000 China; 2 College of Agronomy, Hunan Agricultural University, Changsha, 410128 China

**Keywords:** Fragrant solomonseal, Root rot, Continuous cropping soil, First cropping soil, Low-molecule-weight phenolic acids, Genomic analysis

## Abstract

**Background:**

The root rot of fragrant solomonseal (*Polygonatum odoratum*) has occurred frequently in the traditional *P. odoratum* cultivating areas in recent years, causing a heavy loss in yield and quality. The phenolic acids in soil, which are the exudates from the *P. odoratum* root, act as allelochemicals that contribute to the consecutive monoculture problem (CMP) of the medicinal plant. The aim of this study was to get a better understanding of *P. odoratum* CMP.

**Results:**

The phenolic acid contents, the nutrient chemical contents, and the enzyme activities related to the soil nutrient metabolism in the first cropping (FC) soil and continuous cropping (CC) soil were determined, and the differentially expressed genes (DEGs) related to the regulation of the phenolic acids in roots were analyzed. The results showed that five low-molecule-weight phenolic acids were detected both in the CC soil and FC soil, but the phenolic acid contents in the CC soil were significantly higher than those in the FC soil except vanillic acid. The contents of the available nitrogen, available phosphorus, and available potassium in the CC soil were significantly decreased, and the activities of urease and sucrase in the CC soil were significantly decreased. The genomic analysis showed that the phenolic acid anabolism in *P. odoratum* in the CC soil was promoted. These results indicated that the phenolic acids were accumulated in the CC soil, the nutrient condition in the CC soil deteriorated, and the nitrogen metabolism and sugar catabolism of the CC soil were lowered. Meantime, the anabolism of phenolic acids was increased in the CC plant.

**Conclusions:**

The CC system promoted the phenolic acid anabolism in *P. odoratum* and made phenolic acids accumulate in the soil.

**Supplementary Information:**

The online version contains supplementary material available at 10.1186/s12870-021-03135-x.

## Background

*Polygonatum odoratum* (Mill.) Druce, popularly known as fragrant solomonseal, is a traditional Chinese perennial medicinal plant mainly cultivated in the southern parts of China and the other Southeast Asian countries such as Thailand and Vietnam. As a medicinal and edible plant, its rhizome has the functions of removing dryness, promoting secretion, and quenching thirst [1, 2], and it is widely welcomed especially in the Southeast Asian markets. However, the root rot of *P. odoratum* has occurred frequently in the planting areas in recent years due to the long-term continuous cropping (CC), causing the serious consecutive monoculture problem (CMP) with a sharp decline both in yield and quality. For example, in Shaodong County of Hunan Province, which was the largest traditional high-quality *P*. *odoratum* planting area in China, the medicinal peasants have almost given up the cultivation of *P*. *odoratum* due to this serious replant disease.

Researches have revealed that CMP is mainly caused by three factors covering the imbalance of soil nutrients, the shift in microorganisms towards pathogenic and allelopathic autotoxicity, and the root exudates of secondary metabolites, among which the root exudates are likely to be considered as the main factor to induce the root rot by influencing rhizosphere microbes and soil nutrients [3]. The root exudates include low-molecule-weight substances such as polysaccharides, vitamins, nucleotides, and phenolic acids. Among them, phenolic acids are most likely to be considered as the allelochemicals that can change membrane permeability, inhibit nutrient uptake, and inactivate plant endogenous hormones to influence the normal physiological process [4].

Phenolic acids are a kind of substance containing active organic acids on their aromatic rings that are mainly synthesized by the shikimic acid pathway which provides phenylpropanoids or synthesized by the polyketide pathway in plants which provides simple phenolic acids directly. Shikimic acid is synthesized from 4-phosphate erythritose via the phosphoenolpyruvic acid and pentose phosphate pathway, and then the aromatic amino acids are subsequently used as precursors for the synthesis of phenolic acids [5]. Phenolic acids in plants are products responding to environmental stresses, and they may be involved in the intracellular and intercellular signaling processes as signal transporting substances [6]. When plant roots are infected, the phenolic acid levels in the root cells are promoted to act as antioxidants and inhibit infectious microorganisms directly [7].

Although phenolic acids are of vital importance for the plant’s interaction with the environment, few documents have been published on the relationship between the *P. odoratum* phenolic acids and the root rot disease. An early study showed that the low-molecule-weight substances extracted from the *P. odoratum* rhizosphere soil solutions exhibited an obvious self-poison effect on *P. odoratum* seedling growth and allelopathy effect on the rhizome spore germination rate [8]. Our recent studies have revealed that the *Polygonatum* CMP could be alleviated or eradicated under the rotation regimes or with an aeroponic system [9, 10], and the miRNAs-regulated genes participating in the sugar and phenylpropanoid metabolism have been enriched through the mircoRNA sequencing on the CC and the first cropping (FC) *P. odoratum* [11]. All these results prompted that the root rot in *P. odoratum* was likely related to the phenolic acid metabolism. Other researchers revealed that the root exudates including phenolic acids may not only involve in the signal transduction and hormone synthesis but also modify the rhizosphere soil environment to regulate the microbial community in the rhizosphere in some other medicinal plants such as *Rehmannia glutinosa* [5, 12]. Besides, recent researches have provided more information on the complex correlation among the root rot, phenolic acids, and CMP. Although phenolic acids in the CC soils stimulate the propagation of *Fusarium* which directly results in root rot, single phenolic acid also plays a role as a double-edged sword to decrease the *Fusarium* growth and production at a lower level, and the CC system can strengthen the phenolic acid accumulation in the rhizosphere soils of peanut, *Panax notoginseng*, and *Chrysanthemum morifolium* [13–15].

In this study, the low-molecule-weight phenolic acids from the rhizosphere soil of FC and CC *P. odoratum* were identified, and the relative chemical and physiological indices for evaluating the influences of the phenolic acids on the soil were also determined. Furthermore, the transcriptome profiles from the FC and CC rhizomes of *P. odoratum* were compared through high-throughput sequencing, and then the differentially expressed genes (DEGs) were analyzed. Then the pathway related to the phenolic acid metabolism was analyzed using the Kyoto Encyclopedia of Genes and Genomes (KEGG). This research might provide more information to understand the regulatory function of the phenolic acids in *P. odoratum* under the CC system.

## Results

### Rhizosphere soil phenolic acids

The rhizosphere soil phenolic acids were determined using the high-performance liquid chromatography (HPLC) method as compared to the standard samples of *p*-hydroxybenzoic acid, vanillic acid, syringic acid, cumaric acid, and ferulic acid with the retention time of 15.42, 19.29, 21.84, 27.16, and 28.94 min, respectively (Fig. S1A), and the above five phenolic acids were detected both in the FC and CC rhizosphere soil (Fig. S1B, Fig. S1C). R^2^ of the five regression curves was 0.999 (Table 1). The good linearity indicated that the determination method was reliable. The phenolic acid contents in the FC and CC rhizosphere soil were calculated according to the equations in Table 1. As shown in Table 2, *p*-hydroxybenzoic, syringic acid, cumaric acid, and ferulic acid contents in the CC soil were significantly higher than those in the FC soil, whereas vanillic acid in the CC and FC soil had no significant difference, although vanillic acid contents in the CC and FC soil exhibited much higher level than those of the other phenolic acids. The results showed that the phenolic acid contents in the CC soil were higher than those in the FC soil.

**Table 1:** Regression equations of phenolic acid standard samples using external standard method

Phenolic acid standard sample	Regression equation	R^2^	Retention time (min)
*p*-hydroxybenzoic acid	y = 19.394x + 0.5019	0.999	15.42
Vanillic acid	y = 19.040x + 0.0441	0.999	19.29
Syringic acid	y = 29.678x - 0.0005	0.999	21.84
Cumaric acid	y = 42.687x + 0.3221	0.999	27.16
Ferulic acid	y = 27.663x - 0.0114	0.999	28.94

**Table 2:** Phenolic acid contents in FC and CC rhizosphere soil

Rhizosphere soil sample	*p*-hydroxybenzoic acid (μg/g)	Vanillic acid (μg/g)	Syringic acid (μg/g)	Cumaric acid (μg/g)	Ferulic acid (μg/g)
FC	0.0554	0.2806	0.0337	0.0204	0.0187
CC	0.1372^a^	0.3003	0.0590^a^	0.0385^a^	0.0303^a^

Note: ^a^ was marked for the significant difference within the same column with the T-Student’s method (*P* < 0.05, *n* = 3). FC stands for first cropping, and CC stands for continuous cropping

### Rhizosphere soil chemical properties and enzyme activities

The rhizosphere soil elements of the CC and FC system reflecting the soil nutritional status were determined in this experiment. As shown in Table 3, the contents of total phosphorus (TP), total potassium (TK), available nitrogen (AN), available phosphorus (AP), available potassium (AK) in the CC soil were significantly lower than those in the FC soil, whereas the total nitrogen (TN) contents between the CC soil and FC soil had no significant change. Although TN in the CC soil showed no significant difference as compared to that in the FC soil, AN, which is the form of nitrogen that can be absorbed directly by plants, decreased significantly, and the other nutrient chemicals also decreased significantly. These results insisted that the CC soil nutritional status had deteriorated.

**Table 3:** Chemical properties in FC and CC rhizosphere soil

Rhizosphere soil sample	TN (g/kg)	TP (g/kg)	TK (g/kg)	AN (mg/kg)	AP (g/kg)	AK (mg/kg)
FC	1.953 ± 0.027	0.355 ± 0.015^a^	1.362 ± 0.009^a^	83.650 ± 3.031^a^	0.259 ± 0.027^a^	34.740 ± 1.119^a^
CC	2.038 ± 0.045	0.293 ± 0.015	1.177 ± 0.228	69.650 ± 3.031	0.226 ± 0.052	25.087 ± 1.132

Note: values were expressed as mean ± standard error, and ^a^ was marked for the significant difference within the same column with the T-Student’s method (*P* < 0.05, *n* = 3). FC stands for first cropping, and CC stands for continuous cropping. TN stands for total nitrogen, TP stands for total phosphorus, TK stands for total potassium, AN stands for available nitrogen, AP stands for available phosphorus, and AK stands for available potassium

The rhizosphere soil enzymes related to the soil nutrient metabolism level were also determined in this experiment. As shown in Table 4, the activities of polyphenol oxidase (PPO), catalase (CAT), and acid phosphatase (ACP) had no significant differences between the CC soil and FC soil, whereas the activities of urease (UE) and sucrase (SC) in the CC soil were significantly lower than those in the FC soil. The results indicated that the soil oxidative metabolism and phosphorus utilization had not been changed between the CC soil and FC soil, but the levels of the nitrogen metabolism and sugar catabolism of the CC soil were lowered as compared to the FC soil.

**Table 4:** Soil enzyme activities in FC and CC rhizosphere soil

Rhizosphere soil sample	PPO (mg/d/g)	CAT (μmol/d/g)	UE (μg/d/g)	ACP (μmol/d/g)	SC (mg/d/g)
FC	49.269 ± 0.438	17.867 ± 1.022	392.069 ± 32.173^a^	22.871 ± 0.830	34.800 ± 0.188^a^
CC	47.727 ± 0.688	17.284 ± 1.026	298.174 ± 14.843	22.004 ± 0.321	20.801 ± 0.177

Note: values were expressed as mean ± standard error, and ^a^ was marked for the significant difference within the same column with the T-Student’s method (*P* < 0.05, n = 3). FC stands for first cropping, and CC stands for continuous cropping. PPO stands for polyphenol oxidase, CAT stands for catalase, UE stands for urease, ACP stands for acid phosphatase, and SC stands for sucrase

### RNA-seq analysis and qRT-PCR verification

After filtering out the low-quality sequences, 299,730,812 clean reads, which were accounted for 95.38% of the raw reads, were obtained from the samples of the CC and FC *P. odoratum* roots, and Q20 and Q30 values and GC contents were all in the reliable ranges (Table S1). Owing to the absence of the reference genome, the total clean reads were spliced to obtain 842,213 transcripts and 510,970 unigenes, and the N50 lengths of the transcripts and unigenes were 931 and 1167 bp, respectively, and 298,939 unigenes were annotated in the databases including NR, NT, KO, SwissProt, PFAM, GO, and KOG, whereas 176,156 unigenes were annotated in the GO database. Then a total number of 15,788 DEGs were screened out in the CC vs FC root tissues including 4843 up-regulated DEGs and 10,945 down-regulated DEGs (padj < 0.05).

**Fig. 1:**
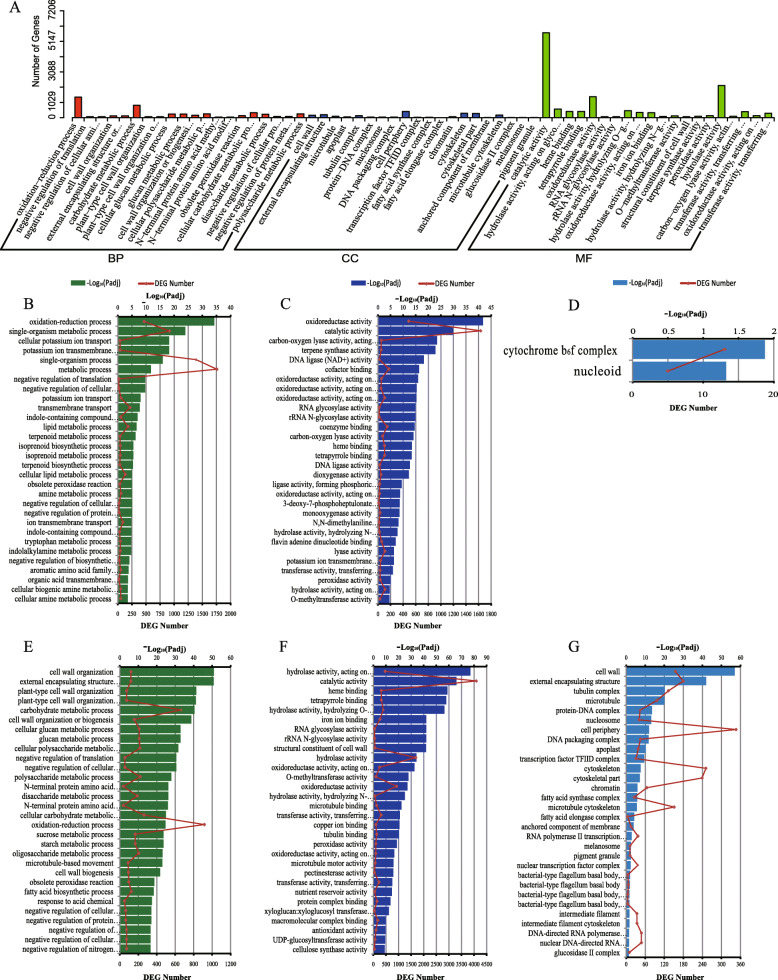
GO enrichments of DEGs in CC vs FC root tissues of *Polygonatum odoratum*. DEGs stands for differentially expressed genes. FC stands for first cropping, and CC stands for continuous cropping. **A**: DEGs in CC vs FC root tissues of *Polygonatum odoratum*, **B** - **D**: up-regulated DEGs in biological process, molecular function, and cellular component, respectively; **E**-**G**: down-regulated DEGs in biological process, molecular function, and cellular component, respectively. In Fig. 1A, BP stands for biological process, CC stands for cellular component, and MF stands for molecular function

These DEGs were significantly enriched in 307 GO functional items. The biological processes included “oxidation-reduction process”, “carbohydrate metabolic process”, “polysaccharide metabolic process”, etc.; the molecular functions included “catalytic activity”, “oxidoreductase activity”, “hydrolase activity”, etc.; the cell components included “cell wall”, “cell periphery”, “cytoskeletal part”, etc. (Fig. 1A). The up-regulated DEGs were mainly enriched in the metabolic process, the single-organism metabolic process, the catalytic activity, the oxidoreductase activity, etc. (Fig. 1B-D); the down-regulated DEGs were mainly enriched in the oxidation-reduction process, the carbohydrate metabolic process, the hydrolase activity (acting on glycosyl bonds), the hydrolase activity, the external encapsulating structure, the cell periphery, etc. (Fig. 1E-G). The results showed that the gene expressions of the CC plant were lower than those of the FC plant on the whole level, but the responses of *P. odoratum* to CMP were regulated by complicated networks, and lots of DEGs took part in the processes such as the secondary metabolism and the response reaction. It should be mentioned that even the up-regulated DEGs and the down-regulated DEGs co-existed in the same processes. For example, although 665 DEGs were significantly down-regulated in the carbohydrate metabolic process, the number of significantly up-regulated DEGs was also up to 177 (Table S2).

**Fig. 2:**
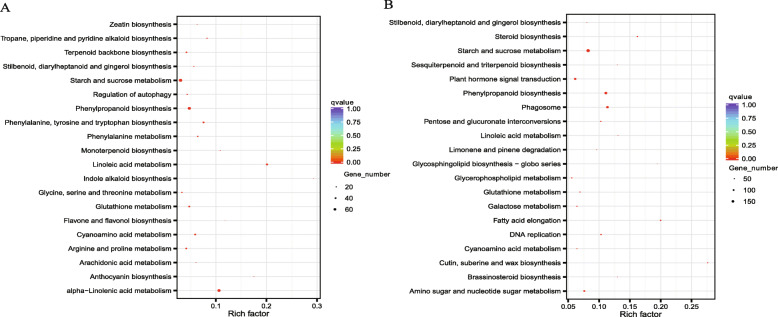
KEGG pathway enrichment of DEGs of CC vs FC root tissues of *Polygonatum odoratum*. DEGs stands for differentially expressed genes. FC stands for first cropping, and CC stands for continuous cropping. **A**: Up-regulated DEGs of CC vs FC root tissues of KEGG pathway enrichment, **B**: down-regulated DEGs of CC vs FC root tissues of KEGG pathway enrichment

The KEGG enrichment analysis showed that DEGs were mainly enriched in the metabolic and biosynthetic pathways including phenylpropanoid biosynthesis, linoleic acid metabolism, phenylalanine metabolism, and starch and sucrose metabolism pathway, etc. The up-regulated DEGs were significantly enriched in the pathways such as “phenylalanine, tyrosine and tryptophan biosynthesis”, “phenylalanine metabolism”, “phenylpropanoid biosynthesis”, and “nitrogen metabolism” (Fig. 2A), whereas the down-regulated DEGs were significantly enriched in the pathways such as “plant hormone signal transduction”, “DNA replication”, “brassinosteroid biosynthesis”, and “sesquiterpenoid and triterpenoid biosynthesis” (Fig. 2B).

**Fig. 3:**
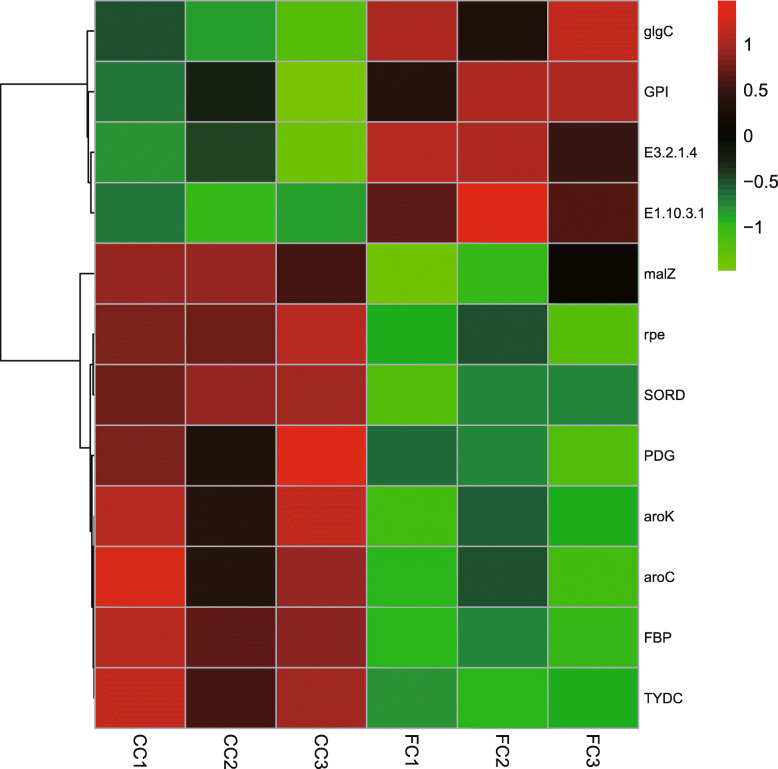
Heatmap of enriched DEGs relative to phenolic acid metabolism in CC vs FC root tissues of *Polygonatum odoratum*. DEGs stands for differentially expressed genes. FC stands for first cropping, and CC stands for continuous cropping (n = 3)

To eliminate those DEGs with relatively lower expressions, log_2_foldchange > 1.5 was selected as the screening condition, the pathways closely relative to the phenolic acid metabolism were enriched including “pentose phosphate pathway”, “pentose and glucoronate interconversions”, “starch and sucrose metabolism”, “tyrosine metabolism, and phenylalanine”, “tyrosine, and tryptophan biosynthesis” (Fig. S2), and then the significantly enriched DEGs were found including *FBP*, *PGD*, *rpe*, *GPD*, *SORD*, *malZ*, *glgC*, *E3.2.1.4*, *aroK*, *aroC*, *TYDC*, and *E1.10.3.1* (Table 5). These DEGs were classified into two groups: down-regulated DEGs and up-regulated DEGs. Among them, *FBP*, *PGD*, *rpe*, *SORD*, *malZ*, *aroK*, *aroC*, and *TYDC* were up-regulated, whereas *glgC*, *GPI*, *E3.2.1.4*, and *E1.10.3.1* were down-regulated (Fig. 3). Among these genes, it was found that the genes that encoded the enzymes in favor of the phenolic acid synthesis were up-regulated, whereas those unfavorable for the phenolic acid synthesis were down-regulated.

**Table 5:** Enriched DEGs relative to phenolic acid metabolism in CC vs FC root tissues of *Polygonatum odoratum*

Pathway	Gene annotation	DEGs	|log_2_foldchange|	Regulation
Pentose phosphate pathway	*FBP*: fructose-1,6-biphospatase [EC:3.1.3.11]	60,288.238790	1.8774	+
		60,288.235250	1.6639	+
	*PGD*: 6-phosphogluconate dehydrogenase [EC:1.1.1.44]	60,288.211711	1.5588	+
	*rpe*: ribulose-phosphate 3-epimerase [EC:5.1.3.1]	60,288.218462	1.9319	+
	*GPI*: glucose-6-phosphate isomerase [EC:5.3.1.9]	60,288.243910	2.0490	–
Pentose and glucoronate interconversions	*SORD*: L-iditol 2-dehydrogenase [EC:1.1.1.14]	60,288.223504	1.8309	+
		60,288.223506	1.6717	+
		60,288.218324	1.7043	+
Starch and sucrose metabolism	*malZ*: α-glucosidase [EC:3.2.1.20]	60,288.201647	3.9725	+
	*glgC*: glucose-1-phosphate adenylyltransferase [EC:2.7.7.27]	60,288.33753	3.1600	–
		60,288.33754	2.7498	–
		60,288.206885	2.1132	–
	*E3.2.1.4*: endoglucanase [EC:3.2.1.4]	60,288.14730	4.4889	–
		60,288.10931	5.5413	–
		60,288.10932	6.4114	–
		60,288.10933	6.1401	–
		60,288.10934	7.8762	–
		60,288.148605	1.8496	–
		60,288.148607	2.5051	–
		60,288.148608	1.9175	–
Phenylalanine, tyrosine and tryptophan biosynthesis	*aroK*: shikimate kinase [EC:2.7.1.71]	60,288.274987	1.6112	+
		60,288.274989	2.4732	+
		60,288.241945	1.9399	+
	*aroC*: chorismate synthase [EC:4.2.3.5]	60,288.232788	1.6419	+
		60,288.230454	1.5993	+
		60,288.239452	1.8285	+
		60,288.239453	1.7613	+
		60,288.239454	1.8178	+
Tyrosine metabolism	*TYDC*: tyrosine decarboxylase [EC:4.1.1.25]	60,288.348666	3.8613	+
		60,288.348669	4.0964	+
		60,288.198712	3.1611	+
	*E1.10.3.1*: polyphenol oxidase [EC:1.10.3.1]	60,288.244462	2.3452	–
		60,288.367167	2.5857	–

Note: DEGs stands for differentially expressed genes. FC stands for first cropping, and CC stands for continuous cropping. + stands for up-regulation, and - stands for down-regulation

**Fig. 4:**
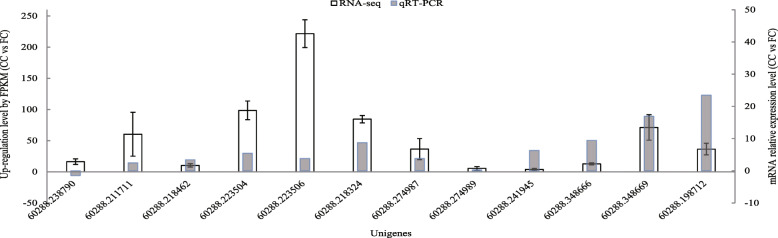
Up-regulated DEGs expression level and their mRNA expression level of CC and FC root tissues. FC stands for first cropping, and CC stands for continuous cropping

To verify the above results, a total of 12 up-regulated DEGs in the CC vs FC plants related to the synthesis of phenolic acids were selected from Table 5 and were tested at the mRNA expression level. The expression levels of DEGs after normalization were presented, and then their mRNA expressions of these DEGs were verified using the quantitative real-time PCR (qRT-PCR). As shown in Fig. 4, the mRNA levels of 11 DEGs were increased except that of 60,288.238790. These results proved that the enriched pathways related to the phenolic acid anabolism through RNA-seq analysis in this study were reliable.

## Discussion

The soil phenolic acids are usually released by plant leaching, root exudation, or decomposition of plant residues [16, 17]. For the low-molecule-weight phenolic acids, there are two groups including benzoic acid derivatives and cinnamic acid derivatives, which play an important role in plant growth and interactions with the environment. These molecules reduce water utilization, hydraulic conductivity, and nutrient uptake to influence the plant’s physiological state [18]. In this study, five low-molecule-weight phenolic acids were identified from the *P. odoratum* rhizosphere soil, i.e., *p*-hydroxybenzoic acid, vanillic acid, syringic acid, cumaric acid, and ferulic acid, where *p*-hydroxybenzoic acid, vanillic acid, and syringic acid were from benzoic acid derivatives, and cumaric acid and ferulic acid were from cinnamic acid derivatives. Although their total contents were very subtle in the soil, the phenolic acid contents in the CC soil were significantly higher than those in the FC soil. These results supported that the CC system might result in an accumulation of phenolic acids secreted by the medicinal plant.

The content level and balance of nitrogen, phosphorus, and potassium in soil are essential for plant growth and development [19]. It was observed that TP, TK, AN, AP, and AK were significantly reduced in the CC soil. Subsequently, this phenomenon might lead to nutrient deterioration in the CC soil for medicinal plant growth. Soil AN is the nitrogen form which is easy to be absorbed and used directly by plants, mainly including NH^+^_4_-N, NO^-^_3_-N, amino acid, amide, and hydrolysable protein nitrogen [20]. The early research had proved that nitrogen in soil inhibits the formation of phenolic acids in plants, and there was a negative correlation between the total phenolic acids and the soil nitrogen content according to the “carbon/nutrient balance hypothesis” [21]. It was also observed this correlation in our experiment. The result showed that the AN in the CC soil was significantly lower than that in the FC soil, whereas the phenolic acids in the CC soil were at a higher level.

The phenolic acids are detrimental to the rhizosphere soil environment and can change the soil microbial community [9]. Phenolic acids have a hydroxyl group and a carboxyl group. For this reason, they can hydrogen-bond with the soil enzymes and change the soil pH value to worsen the soil’s physiological status. The activities of soil enzymes can reflect the soil health status [22]. Soil SC hydrolyzes sucrose into monosaccharides, which are closely related to soil nutrition, whereas soil UE hydrolyzes urea to produce ammonia, which is the source of AN. In this experiment, it was found that the activities of UE and SC in the CC soil were decreased as compared to those in the FC soil. Under this situation, the utilization of sugar and the production of AN were weakened in the CC soil, and then the lack of nitrogen could lead to a comparatively higher content of phenolic acids in the CC plant as compared to that in the FC plant. Consequently, more phenolic acids were secreted and finally accumulated in the CC soil.

**Fig. 5:**
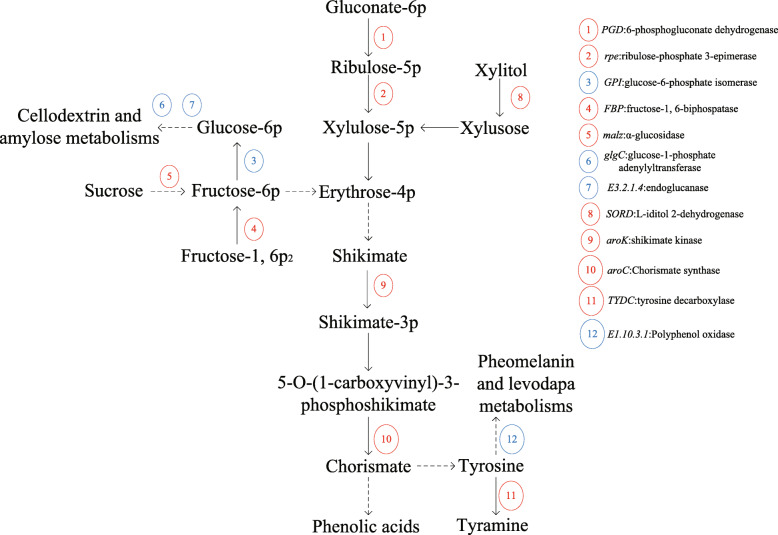
Regulation of phenolic acid metabolism in *P. odoratum* root. Red number stands for up-regulation, and blue number stands for down-regulation

The high-throughput sequencing and relative qRT-PCR analysis also revealed that the gene expressions of the enzymes that catalyzed the key steps in the synthesis of the phenolic acids were regulated in the *P. odoratum* root tissues. The pentose pathway and relative pathways are the major source for metabolic intermediates in plants [23] and can provide erythrose-4-phosphate as a precursor for shikimic acid. In the study, 12 kinds of DEGs that were closely relative to the shikimic acid and chorismate metabolisms were enriched, where eight of them were up-regulated, and four of them were down-regulated (Fig. 5).

Among the up-regulated genes*, PDG*, *rpe*, and *SORD* encoded 6-phosphogluconate dehydrogenase, ribulose-phosphate 3-epimerase, and L-iditol 2-dehydrogenase, respectively, and they were the enzymes for synthesis of ribulose-5-phosphate, xylulose-5-phosphate, and xylusose, respectively [24–26]. The up-regulation of these genes was beneficial to the xylusoe-5-phosphate biosynthesis. *FBP* and *malZ* encoded fructose-1, 6-bisphosphatase and α-glucosidase, respectively. The up-regulation of them was favorable for the synthesis of fructose-6-phosphate, which was material for the synthesis of erthrose-4-phosphate, a precursor of shikmic acid [27, 28]. *aroK* and *aroC* encoded shikimate kinase and chorismate synthase, respectively [29, 30], and the up-regulation of them was beneficial to chorismate biosynthesis, which was the precursor of the low-molecule-weight phenolic acids. On the contrary, the down-regulation of *glgC*, *E3.2.1.4*, and *GPI*, which encoded glucose-1-phosphate adenylyltransferase, endoglucanase, and glucose-6-phosphate isomerase, respectively, could inhibit the cellodexitrin and amylose metabolism [31–33]. This process in turn could provide more materials for the erthrose-4-phosphate biosynthesis. Early researches have proved that the phenolic acid biosynthesis in plants is triggered by biotic and abiotic stresses [34, 35]. Likewise, the results in our study also supported that the biosynthesis of phenolic acids in the CC plant proceeded through the activation of the primary pathway (shikimic acid) and the suppression of the relative branch pathway (cellodexitrin and amylose).

Tyramine is the product of tyrosine decarboxylation, and partial phenolic acids in plants can be conjugated with tyramine especially when the plant is under stress [36, 37]. The tyramine-conjugated phenolic compounds act as phytoalexins to make plants adapt to the external stress, and the biosynthetic processes including the shikimate, phenylpropanoid, and arylmonoamine pathways are activated [38]. The conjugation is also favorable for more phenolic acids to accumulate. In this experiment, it was also found that the expression of tyrosine decarboxylase in the CC plant was up-regulated and polyphenol oxidase was down-regulated. It was speculated that the change might help to promote the phenolic acid anabolism in *P. odoratum* in the CC soil and made them accumulate in the soil.

The inducement of the root rot of *P. odoratum* in the CC soils was very complicated. The multiple factors to cause CMP may mainly include soil element imbalance, soil microbial population transforming to harmful population, and autotoxicity caused by the secretions from plant roots, but the comprehensive mechanism for CMP remains unclear [3]. For *P. odoratum* in the CC soils, the nutrient composition and the soil enzymes promoted the synthesis of phenolic acids. On the other hand, there might be multiple gene families activated together to accelerate the phenolic acid synthesis process coordinately to influence the CMP formation.

## Conclusions

In this study, five low-molecule-weight phenolic acids were identified from the *P. odoratum* rhizosphere soil. Among them, the phenolic acid contents in the CC soil were significantly higher than those in the FC soil except vanillic acid. The contents of AN, AP, and AK in the CC soil were significantly higher than those in the FC soil, and the nitrogen metabolism and sugar catabolism of the CC soil were lowered. The genomic analysis showed that the phenolic acid anabolism in *P. odoratum* in the CC soil was promoted. This research presented us with a preliminary understanding of the role of phenolic acids in the CMP of *P. odoratum*. In perspective, proteomics and metabolomics are needed to look into more metabolic mechanism details of phenolic acid metabolism on the root rot of *P. odoratum*.

## Methods

### Experiment materials and chemicals

The *P. odoratum* cultivar “Zhushiwei” was selected as the experimental material and authenticated by associate Professor Zefa Liu, a horticulturist from Loudi Agricultural Institute, Hunan Province, China. The roots and rhizosphere soil of *P. odoratum* were collected in May, 2017 from the Gutang Town Experimental Station of Loudi City, Hunan Province, China with the permission of Loudi Agricultural Institute, Hunan Province, China. For the CC system, *P. odoratum* was cultivated in the land where the same plants had been harvested; for the FC system, *P. odoratum* was cultivated on the same date in the land near CC where the cabbages had been harvested. The collection of rhizosphere soil was according to Riley and Barber’s method [39, 40]. Briefly, the soil with a complete root system was excavated in the selected plot, the large soil without root was shaken off gently, and then the soil adhered to the root circumference (0–5 mm from the root circumference) was collected with a brush as the rhizosphere soil. The rhizosphere soil was air-dried under room temperature, passed through the 2 mm sieve, and stored at 4 °C until use.

Methanol, *p*-hydroxybenzoic acid, vanillic acid, syringic acid, cumaric acid, ferulic acid, and methanol were of chromatographic purity, and the other chemicals were of analytical purity.

### Rhizosphere soil phenolic acid determination

The rhizosphere soil of 1.0 g was added with 3.0 mL 1 N NaOH, and then shaken at 4 °C overnight. After centrifuged with 8000×g at 4 °C for 10 min, the supernatant was adjusted to pH 2.5 with HCl. After the liquid was extracted two times with 10 mL ethyl acetate, it was concentrated to dryness under nitrogen, and then dissolved in methanol. The solution was filtered with a 0.45 μm membrane for the HPLC analysis.

The HPLC conditions were as follows: volume of 10 μL was loaded on a Kromasil C_18_ column (250 mm × 4.6 mm × 5 μm) (Akzo Nobel, Amsterdam, Netherlands) with a Rigol HPLC L3000 (Rigol Technologies, Beijing, China). The gradient elution was with eluent A 1% phosphoric acid and eluent B methanol. The elution gradients were 80% A + 20% B on 0–10 min, 70% A + 30% B on 10–20 min, 50% A + 50% B on 20–30 min, 50% A + 50% B on 30–40 min, 80% A + 20% B on 40–45 min, and 80% A + 20% B on 45–55 min, respectively, with the rate of 1 mL/min at 30 °C, and the signal was detected at 280 nm with an ultraviolet detector. For the standard curve, *p*-hydroxybenzoic acid, vanillic acid, syringic acid, cumaric acid, and ferulic acid were used as standard samples and detected as above. The external standard method was used for the quantitative analysis of the phenolic acids as per Wang et al. [41].

### Determination of rhizosphere soil chemical properties and enzyme activity

The TN content of the rhizosphere soil was determined with the Kjeldahl method, the TP content of the rhizosphere soil was determined with the molybdenum-blue colorimetry after the soil was extracted with the mixture of concentrated sulfuric acid and perchloric acid, the TK content of the rhizosphere soil was determined with the flame spectrophotometry after the soil was extracted with the mixture of concentrated sulfuric acid and perchloric acid, the AN content of the rhizosphere soil was determined with the diffusion method after hydrolysis with the alkaline solution, the AP content of the rhizosphere soil determined with the molybdenum-blue colorimetry after the soil was extracted with sodium hydroxide, and the AK content of the rhizosphere soil was determined with the flame spectrophotometry after the soil was extracted with sodium acetate [42]. The PPO activity of the rhizosphere soil was determined with the catechol method [43]. The CAT activity of the rhizosphere soil was determined as per Kraus and Fletcher [44]. The UE activity of the rhizosphere soil was determined with the indophenol blue colorimetric method [45]. The ACP of the rhizosphere soil activity was determined as per Saa et al. [46]. The SC activity of the rhizosphere soil was determined as per Li and Lu [47]. All of these analyses were assigned using the kits of Suzhou Comin Biotechnology Co. Ltd. and were repeated three times at the biological sample level.

### RNA-seq and bioinformatics analysis of root

The root samples of CC and FC plants were obtained randomly from the fields during the rhizome expansion stage, and each tissue had three biological repeats in total. The library preparation and sequencing were performed in the Novogene Technology Co., Ltd. (Beijing, China). The total RNA was extracted using the Invitrogen Kit (Thermo Fisher Scientific, Beijing, China) and detected with a nanophotometer (Implen Inc., CA, USA). The sequencing libraries were generated using NEBNext Ultra RNA Library Prep Kit for Illumina (New England Biolabs, MA, USA). The libraries were sequenced on Illumina Hiseq Platform and then 150 bp paired-end reads were generated.

The clean reads were obtained from the raw pair-end reads of mRNA sequencing data by removing adapters, low-quality reads, and the reads in which bases could not be determined, and then Trinity was employed to splice the clean-reads to obtain the de novo transcriptome assembly [48]. For DEGs analysis, DESeq R package(1.10.1)was used to screen the differential expression (*n* = 3) with the gene symbol annotation (padj < 0.05) [49]. The KEGG pathway enrichment analysis was performed using KOBAS (2.0) and the pathway with false discovery rate (FDR) ≤ 0.05 was defined as the significantly enriched pathway with DEGs [50].

### qRT-PCR analysis

The total RNA from the *P. odoratum* root was extracted with Trizol reagent according to the manufacture’s instruction. To obtain cDNA, the total RNA (0.5 μg) was reversely transcribed using a TUREscript cDNA Synthesis Kit (Aidlab Biotechnologies Co., Ltd., Beijing, China) with oligo dT as a primer. The candidate genes and the reference gene were analyzed with a qTOWER2.2 real-time PCR (Analytik Jena AG, Jena, Thuringia, Germany) with the primers in Table S3. The protocol for qRT-PCR was as follows: 3 min at 95 °C, followed by 10 s at 95 °C and 30 s at 58 °C with 39 cycles, and then was ended after a melt curve analysis from 60 °C to 95 °C with an increment of 1 °C for 4 s at each step. Three biological replicates were used for each analysis and the data were analyzed using the 2^−ΔΔCt^ method.

### Data statistics

All the experiments were repeated three times at the biological sample level, and the differences were analyzed using IBM SPSS 19.0 (International Business Machines Corporation, Armonk, NY, USA) with the T-student’s method (*P* < 0.05).

## Supplementary Information

**Additional file 1: Fig. S1.** Rhizosphere soil phenolic acid detection with HPLC. FC stands for first cropping, and CC stands for continuous cropping. A: standard samples, B: FC soil, C: CC soil. 1: *p*-hydroxybenzoic, 2: vanillic acid, 3: syringic acid, 4: cumaric acid, 5: ferulic acid.

**Additional file 2: Table S1.** Data output quality summary.

**Additional file 3: Table S2.** Up- and down-regulated DEGs in CC vs FC root tissues.

**Additional file 4: Fig. S2.** Enriched pathways relative to phenolic acid synthesis.

**Additional file 5: Table S3.** Primers designed for qRT-PCR.

**Additional file 6:** Data of up-regulated DEGs expression level and qRT-PCR verification.

**Additional file 7:** Data of soil enzyme activities in FC and CC rhizosphere soil.

**Additional file 8:** Data of phenolic acid contents in FC and CC rhizosphere soil. Typical curve of standard samples. Typical curve of CC soil. Typical curve of FC soil.

**Additional file 9:** Data of heatmap of DEGs relative to phonelic acid metabolism in cc vs fc root.

**Additional file 10:** Data of chemical properties in FC and CC rhizosphere soil.

## Data Availability

All data supporting the findings were contained in the manuscript and its supplementary files except the RNA-seq raw data. And all the RNA-seq raw data were uploaded in the SRA of NCBI (PRJNA507291).

## Abbreviations

ACP: Acid phosphatase
AN: Available nitrogen
AP: Available phosphorus
AK: Available potassium
CAT: Catalase
CC: Continuous cropping
CMP: Consecutive monoculture problem
DEGs: Differentially expressed genes
FC: The first cropping
KEGG: The Kyoto Encyclopedia of Genes and Genomes
HPLC: High-performance liquid chromatography
PPO: Polyphenol oxidase
qRT-PCR: quantitative real-time PCR
SC: Sucrase
TK: Total potassium
TP: Total phosphorus
TN: Total nitrogen
UE: Urease
