# Supplementary material for: Physiological and transcriptomic analyses to reveal underlying phenolic acid action in consecutive monoculture problem of Polygonatum odoratum
Source: BMC Plant Biol. 2021 Aug 7;21:362. doi: 10.1186/s12870-021-03135-x (PMC8349006; doi:10.1186/s12870-021-03135-x)
Supplement: Supplementary file 1 — Additional file 1: Fig. S1. Rhizosphere soil phenolic acid detection with HPLC. FC stands for first cropping, and CC stands for continuous cropping. A: standard samples, B: FC soil, C: CC soil. 1: p-hydroxybenzoic, 2: vanillic acid, 3: syringic acid, 4: cumaric acid, 5: ferulic acid. [file 12870_2021_3135_MOESM1_ESM.docx]

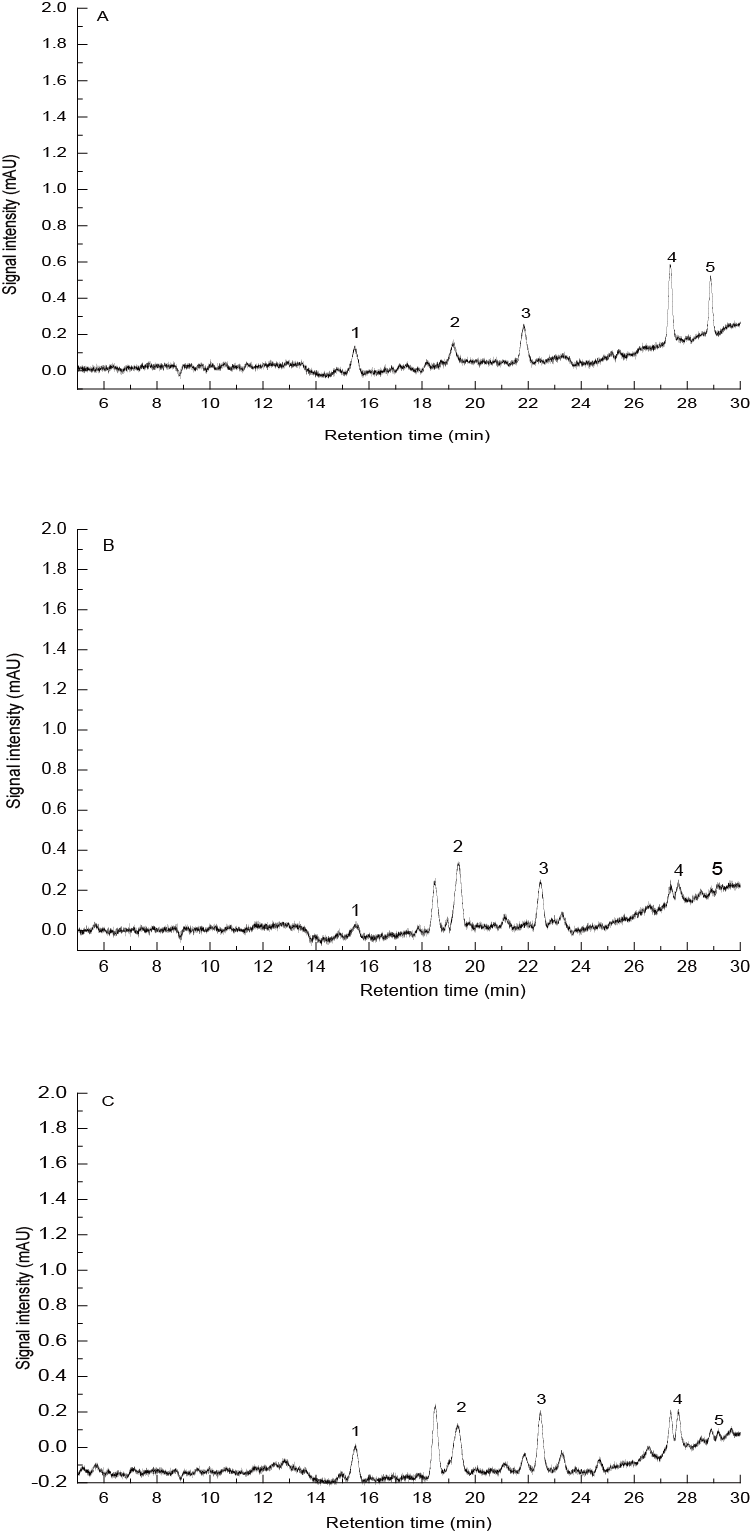


**Figure S1** Rhizosphere soil phenolic acid detection with HPLC. FC stands for first cropping, and CC stands for continuous cropping. A: standard samples, B: FC soil, C: CC soil. 1: *p*-hydroxybenzoic, 2: vanillic acid, 3: syringic acid, 4: cumaric acid, 5: ferulic acid.
