# Supplementary material for: Physiological and transcriptomic analyses to reveal underlying phenolic acid action in consecutive monoculture problem of Polygonatum odoratum
Source: BMC Plant Biol. 2021 Aug 7;21:362. doi: 10.1186/s12870-021-03135-x (PMC8349006; doi:10.1186/s12870-021-03135-x)
Supplement: Supplementary file 2 — Additional file 2: Table S1. Data output quality summary. [file 12870_2021_3135_MOESM2_ESM.docx]

**Table S1**. Data output quality summary

| Sample | Raw reads | Clean reads | Clean bases (G) | Error (%) | Q20 (%) | Q30 (%) | GC (%) |
| --- | --- | --- | --- | --- | --- | --- | --- |
| CC1 | 47966204 | 46487560 | 6.97 | 0.02 | 96.62 | 91.77 | 47.52 |
| CC2 | 47557350 | 45361532 | 6.80 | 0.01 | 97.50 | 93.62 | 47.74 |
| CC3 | 52217002 | 49735868 | 7.46 | 0.01 | 97.51 | 93.63 | 47.59 |
| FC1 | 53369156 | 51386920 | 7.71 | 0.02 | 96.53 | 91.55 | 48.60 |
| FC2 | 48231162 | 45233942 | 6.79 | 0.02 | 96.56 | 91.62 | 48.29 |
| FC3 | 64915492 | 61524990 | 9.23 | 0.02 | 96.50 | 91.48 | 48.20 |

Note: FC stands for first cropping, and CC stands for continuous cropping.
