# Supplementary material for: Physiological and transcriptomic analyses to reveal underlying phenolic acid action in consecutive monoculture problem of Polygonatum odoratum
Source: BMC Plant Biol. 2021 Aug 7;21:362. doi: 10.1186/s12870-021-03135-x (PMC8349006; doi:10.1186/s12870-021-03135-x)
Supplement: Supplementary file 3 — Additional file 3: Table S2. Up- and down-regulated DEGs in CC vs FC root tissues. [file 12870_2021_3135_MOESM3_ESM.docx]

**Table S2.** Up- and down-regulated DEGs in CC vs FC root tissues

| Go term | Description | Type | DEGs (up) | DEGs (down) |
| --- | --- | --- | --- | --- |
| GO:0055114 | Oxidation-reduction process | Biological_process | 467 | 916 |
| GO:0005975 | Carbohydrate metabolic process | Biological_process | 177 | 665 |
| GO:0071944 | Cell periphery | Cellular component | 77 | 346 |
| GO:0003824 | Catalytic activity | Molecular function | 1635 | 2456 |
| GO:0016491 | Oxidoreductase activity | Molecular function | 488 | 924 |
