# Supplementary figures and images for: Physiological and transcriptomic analyses to reveal underlying phenolic acid action in consecutive monoculture problem of Polygonatum odoratum
Source: BMC Plant Biol. 2021 Aug 7;21:362. doi: 10.1186/s12870-021-03135-x (PMC8349006; doi:10.1186/s12870-021-03135-x)

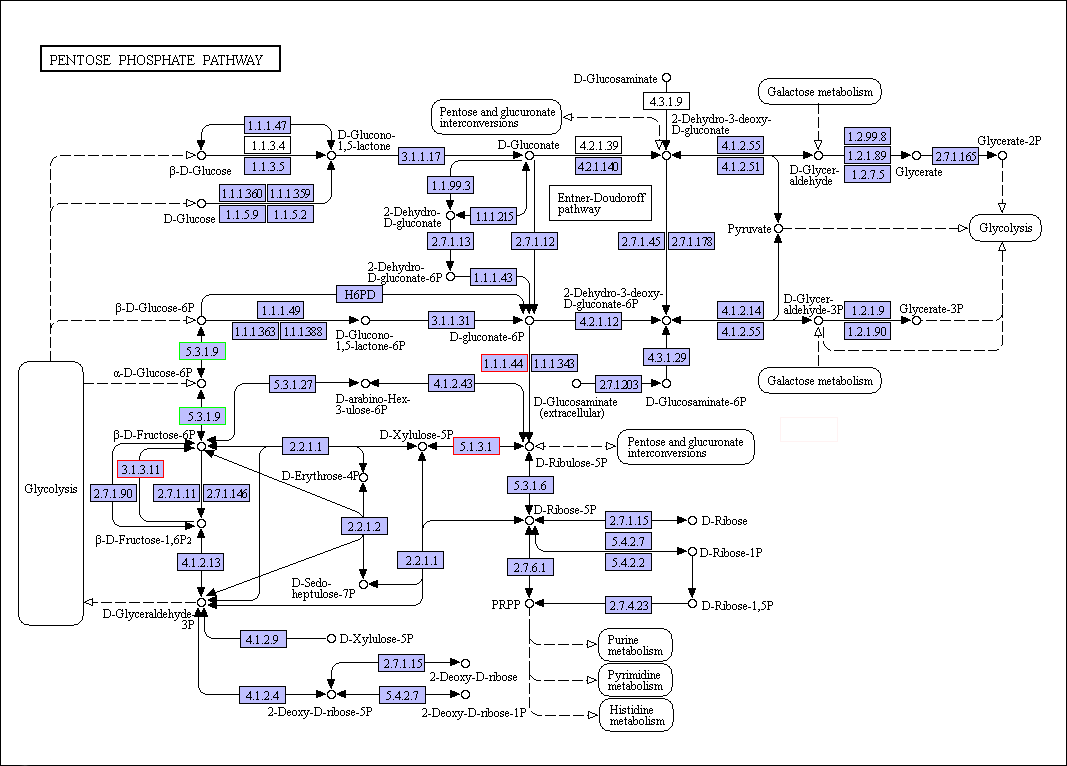


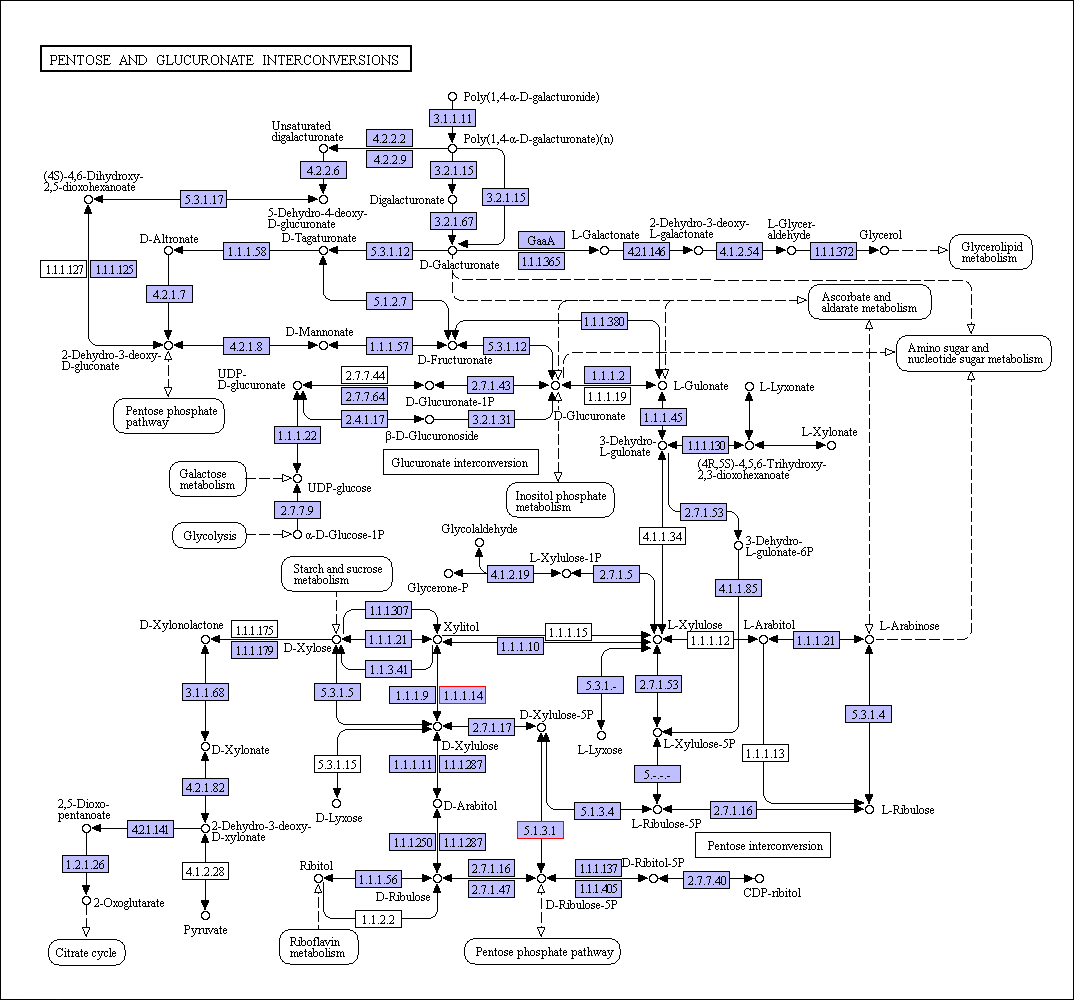


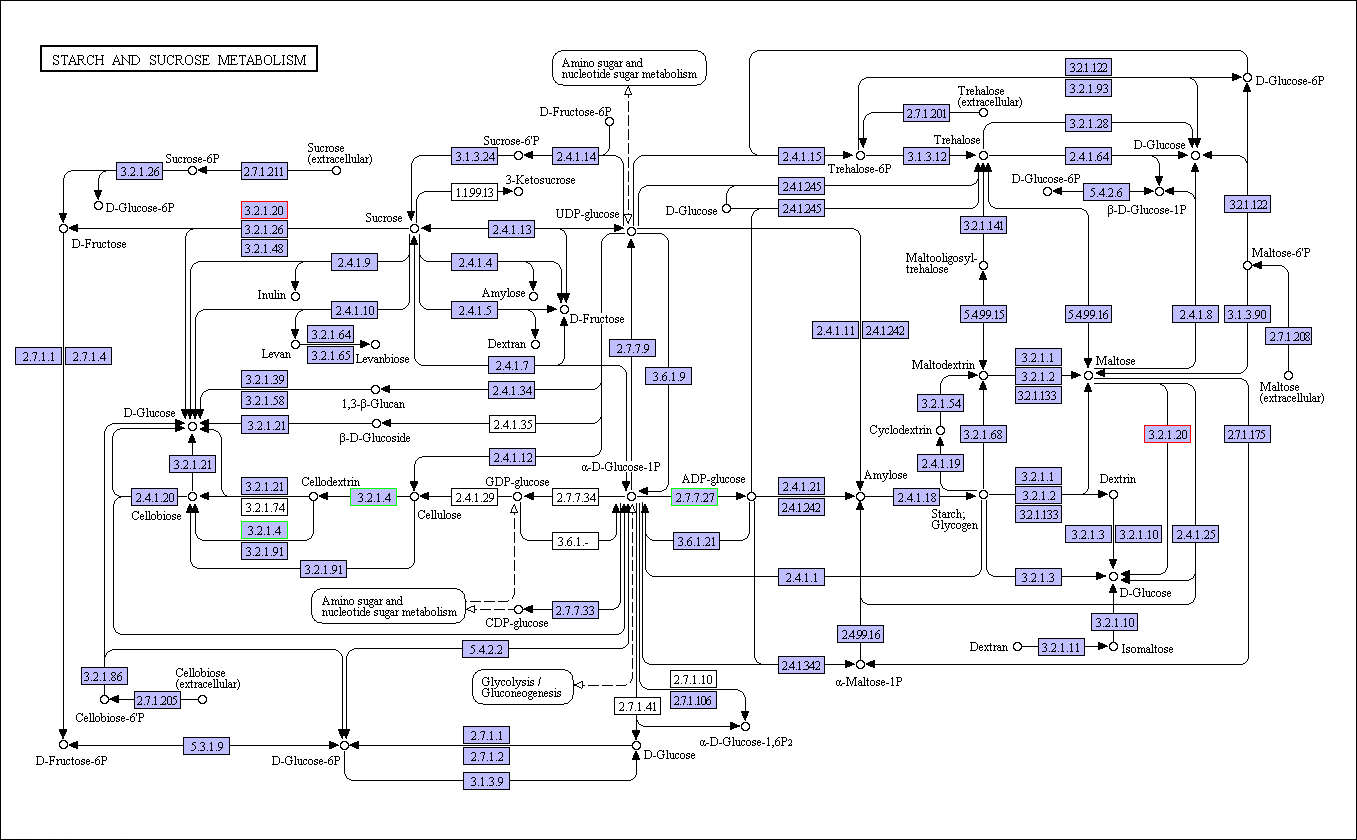


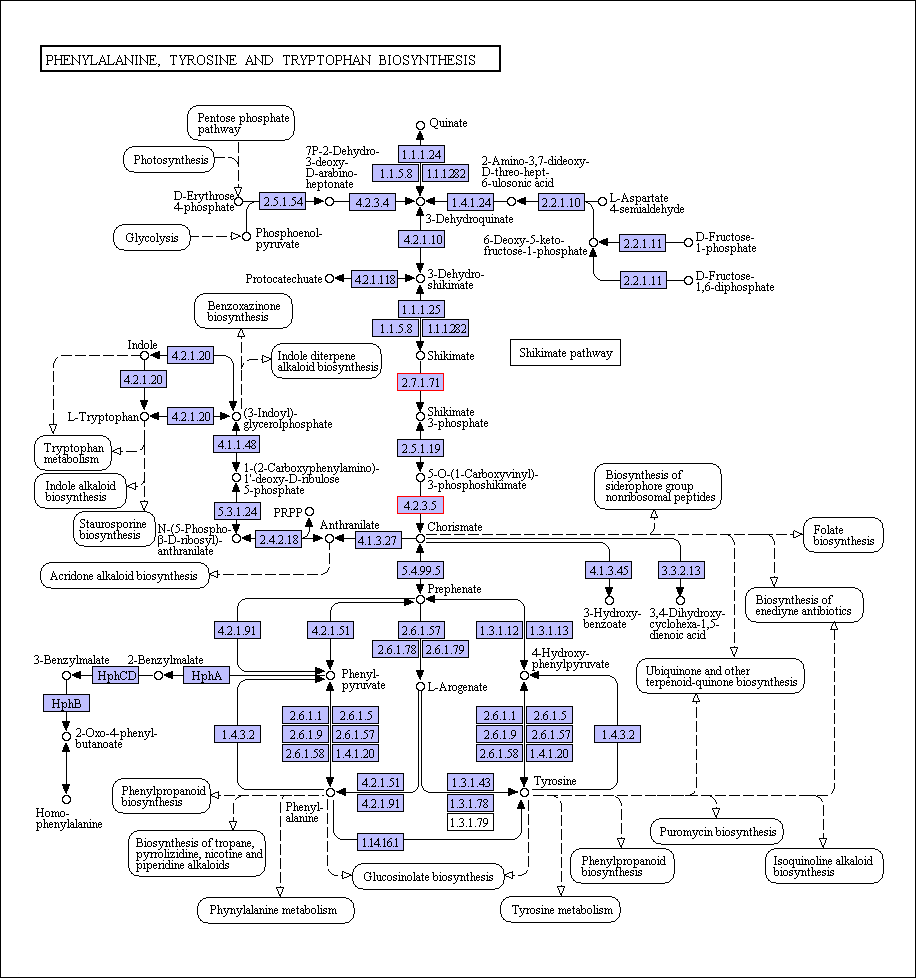


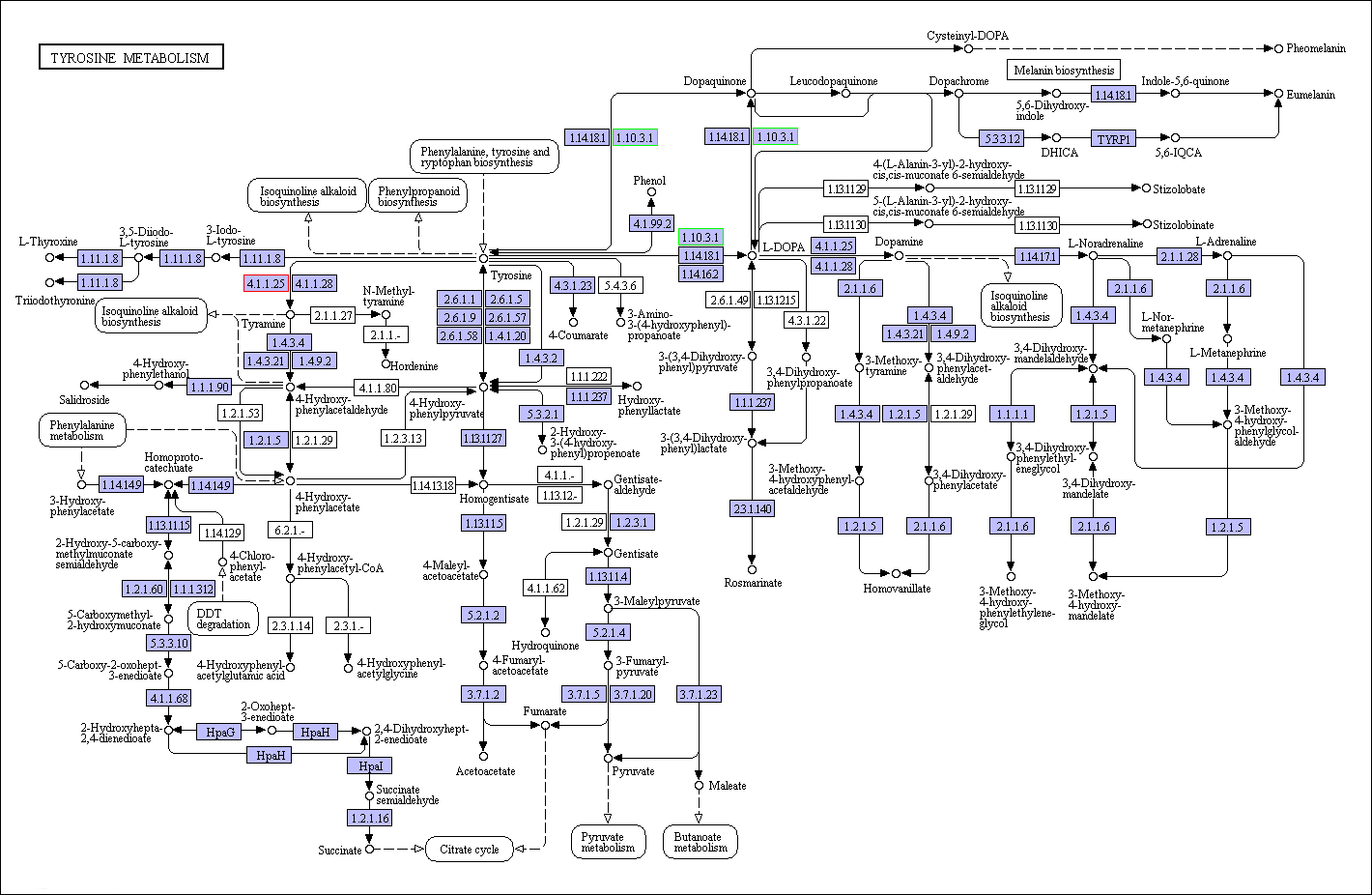


**Figure S2** Enriched pathways relative to phenolic acid synthesis

Supplement: Supplementary file 4 — Additional file 4: Fig. S2. Enriched pathways relative to phenolic acid synthesis. [file 12870_2021_3135_MOESM4_ESM.docx]
