# Supplementary material for: Physiological and transcriptomic analyses to reveal underlying phenolic acid action in consecutive monoculture problem of Polygonatum odoratum
Source: BMC Plant Biol. 2021 Aug 7;21:362. doi: 10.1186/s12870-021-03135-x (PMC8349006; doi:10.1186/s12870-021-03135-x)
Supplement: Supplementary file 5 — Additional file 5: Table S3. Primers designed for qRT-PCR. [file 12870_2021_3135_MOESM5_ESM.docx]

**Table S3.** Primers designed for qRT-PCR

| Sequence name | Primer sequence (5’-3’) |
| --- | --- |
| 60288.238790 | f-CTCTTGATCCGTCTCTTG |
| 60288.238790 | r-CATTGCCTTCGTTTACAG |
| 60288.211711 | f-CGCTCCATTATCTCCATTG |
| 60288.211711 | r-CAAGAAGGCATACGACAG |
| 60288.218462 | f-GTTCGTCTTCTTCCTCTC |
| 60288.218462 | r-GATAACCTCCTCCTCCTC |
| 60288.223504 | f-GGCATATTCCGCTACAAG |
| 60288.223504 | r-TTCTTCTACTTCCGTCTGT |
| 60288.223506 | f-ATAATGAACATCACTGCC |
| 60288.223506 | r-GGTATCAACACTCTCAAG |
| 60288.218324 | f-AAAGGTGGACGATACAAC |
| 60288.218324 | r-AATCAGCAGGATGGACTA |
| 60288.274987 | f-AGACTGTTATCCCTTGTTTC |
| 60288.274987 | r-TGAGCCGATTAGTTGTTG |
| 60288.274989 | f-GTCACTCTCACTGTCTCTG |
| 60288.274989 | r-AGGCTGTTGGAATGTCTT |
| 60288.241945 | f-CACTTCCGATTCTGATTCTT |
| 60288.241945 | r-CGTTCCGTATCCATCTCT |
| 60288.348666 | f-AGAATCCATTGAGACCAT |
| 60288.348666 | r-GAAATAGGCGAAATAGTTG |
| 60288.348669 | f-GGTCAACGCTCTGTCAAC |
| 60288.348669 | r-TGCCAGTCCTTGTAGTCC |
| 60288.198712 | f-GGTCAACGCTCTGTCAAC |
| 60288.198712 | r-TGCCAGTCCTTGTAGTCC |
| 18S | f-CAACCATAAACGATGCCGA |
| 18S | r-AGCCTTGCGACCATACTCC |
